# Examining the Time to Diagnosis in Idiopathic Intracranial Hypertension Presentations in a Specialist Eye Emergency Department

**DOI:** 10.7759/cureus.76550

**Published:** 2024-12-28

**Authors:** Elliott Cope, Carmel Crock, Claude Fahrer

**Affiliations:** 1 Emergency Medicine, Royal Melbourne Hospital, Melbourne, AUS; 2 Emergency, Royal Victorian Eye and Ear Hospital, Melbourne, AUS

**Keywords:** diagnostic delay, idiopathic intracranial hypertension, neurology, neuro-ophthalmology, ophthalmology

## Abstract

Idiopathic intracranial hypertension (IIH) is a neurological disorder characterized by chronic headaches, cognitive difficulties, reduced quality of life, and rarely irreversible visual loss. Community diagnosis is often challenging due to unfamiliarity with current guidelines and a lack of clinical experience, leading to misdiagnosis and treatment delays, which can negatively impact visual recovery and quality of life. Our study examined the time to diagnosis and investigated the barriers to timely diagnosis in adults with newly diagnosed IIH. This retrospective, single-centre cohort study was performed at an Australian quaternary specialised ophthalmology/otolaryngology hospital. Patient data were retrieved from the emergency department (ED) electronic database for the study period ranging from September 1, 2022, to September 1, 2023, for 51 adults with a new diagnosis of IIH. The mean time to ED presentation from symptom onset was 132.2 days (SD = 283.8, range = 1-1767). Of the patients, 55% (28/51) presented to the ED within one month of symptom onset, 20% (9/51) within three months, and 25% (13/51) after three months. The mean time to final diagnosis from ED discharge was 31.2 days (SD = 28.6, range = 1-140). Furthermore, the final diagnosis was achieved for 65% (33/51) in one month, and 90% (46/51) in two months. The mean time to diagnosis from symptom onset was 163.3 days (SD = 312.3, range = 11-1800). Diagnosis of IIH can be difficult and is often delayed, usually due to a lag in being reviewed by an appropriate eye specialist. Our study highlights that a referral pathway to a specialist neuro-ophthalmology centre can result in a timely and accurate diagnosis for individuals suffering from IIH.

## Introduction

Idiopathic intracranial hypertension (IIH) is a neurological condition that often leads to chronic headaches, cognitive issues, lowered quality of life, and, in rare cases, irreversible visual loss [1]. Diagnosing IIH necessitates a multifaceted evaluation encompassing a detailed medical history, a comprehensive neuro-ophthalmological examination, advanced neuroimaging techniques, and lumbar puncture (LP) to measure cerebrospinal fluid pressure [2]. Diagnosis of IIH can be a challenge in the community due to clinicians being unfamiliar with current diagnostic and management guidelines as well as a lack of clinical experience with this condition. This can lead to misdiagnosis and a potential delay in treatment [3]. Research on the average time to diagnosis in IIH is limited with no large studies investigating the time to diagnosis in adults with IIH.

Delays in diagnosis can be detrimental to patients with delays in treatment initiation being associated with poorer visual recovery, permanent visual loss (in the context of fulminant IIH), and a decrease in quality of life attributed to chronic headaches and visual disturbances [4-6]. Prior research has investigated factors that impact the time to a final diagnosis of IIH in an adolescent population [7]. However, to date, similar research has not been conducted in adults. The purpose of this retrospective study is to provide a current outlook on the time to diagnosis and investigate the barriers to timely diagnosis in adults with newly diagnosed IIH who presented to an Australian quaternary eye hospital.

## Materials and methods

Study location

This retrospective, single-centre cohort study was performed at the Royal Victorian Eye and Ear Hospital (RVEEH), Australia, a specialised ophthalmology/otolaryngology hospital. The referral centre serves the state of Victoria with a population of 2.4 million people. There is 24/7 on-call availability of a neuro-ophthalmologist, and a high proportion of ophthalmologic emergencies present to RVEEH either by healthcare practitioner referral or self-presentation in comparison to general emergency departments (EDs).

Patient selection and data retrieval

Data were collected from the electronic patient database at the RVEEH for individuals who were labelled with a ‘working diagnosis of IIH’ upon ED discharge. A working diagnosis of IIH was coded into the electronic medical records from ED after consultation with an on-call neuro-ophthalmologist if the patient met aspects of the modified Dandy criteria. Specifically, signs and symptoms of IIH, normal neurological and mental state exam (except for a 6th nerve palsy and papilloedema), normal neuroimaging (except for features of IIH), and no other cause found [2,8]. Due to the limitations of obtaining a lumbar puncture with opening pressure (LP with OP) on the day of presentation within this specialised ED, the remaining aspects of the modified Dandy criteria (lumbar puncture opening pressure > 25 cm H2O and normal cerebral spinal fluid contents) were not used when defining a case as a ‘working diagnosis of IIH’. After patients with a working diagnosis of IIH were seen in the ED, they were then referred for an outpatient CT-guided LP, further neuro-imaging (an MRI if not obtained on the day of presentation to the ED), and follow-up in a neuro-ophthalmology clinic within one month. A final diagnosis was provided to the patient during their clinic review by a neuro-ophthalmologist utilising the fully modified Dandy criteria. Their subsequent clinic reviews were also audited to ascertain the final diagnosis and the time to final diagnosis from symptom onset. During the period from September 1, 2022, to September 1, 2023, there were 42,891 triaged patients in the ED. A total of 162 (0.38%) patients were identified as having a working diagnosis of IIH.

Exclusion criteria

**Figure 1:**
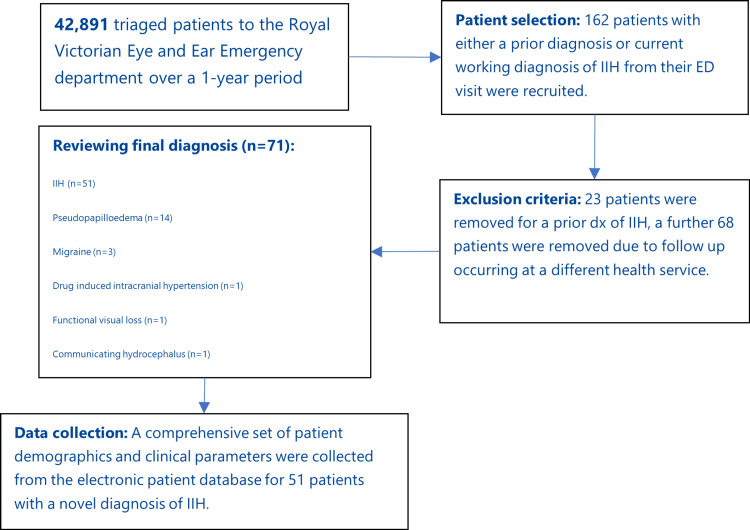
Flowchart of patient selection and data analysis, including the final diagnosis breakdown of patients once reviewed at the outpatient clinic. IIH: idiopathic intracranial hypertension; dx: diagnosis.

Individuals with a documented prior history of IIH were excluded from the analysis. This exclusion resulted in the removal of 23 encounters from the final dataset. Additionally, 68 patients who were not reviewed at the RVEEH outpatient clinics were also excluded as we were unable to gain access to their clinic notes. Finally, 20 patients did not have a final diagnosis of IIH. As a result, the study yielded 51 patients with a novel diagnosis of IIH. The process of patient selection can be seen in Figure 1.

Data collection

A comprehensive set of patient demographics and clinical parameters were collected from ED records, radiology and pathology reports, and clinic notes. This included source of referral, gender, age, history of visual disturbance (diplopia, blurred vision, loss of peripheral vision, loss of central vision), presence of headache, visual acuity (VA) worse than 6/9 corrected in at least one eye, BMI, papilloedema on examination, and final diagnosis. The type and timing of neuroimaging of patients were also recorded and separated into CT brain venogram (CTBV) or MRI and prior to ED, in ED, or outpatient, respectively. LPs were also categorised in this manner.

Temporal features in the diagnostic process, i.e., time to MRI as an outpatient, time to LP as an outpatient, time to ED presentation from symptom onset, time to diagnosis from symptom onset, and time to diagnosis from ED discharge, were all measured in days. Time to CT while in ED was measured in hours. Time to diagnosis was separated into time from patient reporting symptom onset and time to diagnosis post ED discharge.

All measures, which included temporal data points after discharge from ED, i.e., time to MRI as an outpatient, time to LP as an outpatient, and time to diagnosis from ED discharge, used the day of ED presentation as day zero. Time to diagnosis from symptom onset used the date of first symptom complaint (if provided by the patient) as day zero; otherwise, day zero was conservatively estimated as the Saturday of the week when patients described symptom onset. The date of final diagnosis was calculated as the date the patient received their diagnosis at the neuro-ophthalmology clinic.

Data analysis

Descriptive data analysis was performed for all patient demographics, clinical parameters, and temporal features. Data analysis was performed using STATA version 15.1 (StataCorp LLC, College Station, TX) [9].

## Results

Demographics

There was a strong female predominance among the patient group (90%, 46/51). The mean age at final diagnosis was 29.4 (SD = 5.7). A total of 82% (42/51) of individuals were classified as overweight (BMI ≥ 25). This was further stratified with 73% (37/51) of individuals being obese (BMI ≥ 30), and 4% (2/51) classified as morbidly obese (BMI ≥ 40). Headache and visual disturbance (encompassing diplopia, blurry vision, or peripheral/central vision loss) were the most common symptoms at ED presentation (88% and 58%, respectively). Of the patients, 12% (6/51) exhibited reduced visual acuity worse than 6/9 in at least one eye. All patients (51/51) had papilloedema identified on fundoscopy in the emergency department. Referral sources were available for all patients. Of the patients, 86% were referred to our institution by optometrists after having bilateral optic disc oedema identified. Of the patients, 4% (2/51) were referred by private ophthalmologists for concerns regarding IIH, 6% (3/51) were transferred from other hospitals for visual disturbances, and 4% of patients (2/51) self-presented. Patient demographics and referral sources are presented in Table 1.

**Table 1:** Clinical demographics of patients with a novel diagnosis of IIH. IIH: idiopathic intracranial hypertension; BMI: body mass index; VA: visual acuity.

	Number	Percentage (%)
Sex		
Female	46	90
Referral source		
Optometrist	44	86
Ophthalmologist	2	4
Other hospital	3	6
Self-present	2	4
Visual disturbance reported (diplopia, blurred vision, visual loss)		
Yes	30	58
Visual acuity reduction in at least one eye (VA < 6/9)		
Yes	6	12
Weight status		
Overweight (BMI ≥ 25)	42	82
Obese (BMI ≥ 30)	37	73
Morbidly obese (BMI ≥ 40)	2	4
Headache on presentation		
Yes	45	88
Papilloedema on examination		
Yes	51	100

Diagnostic procedures and time to final diagnosis

**Figure 2:**
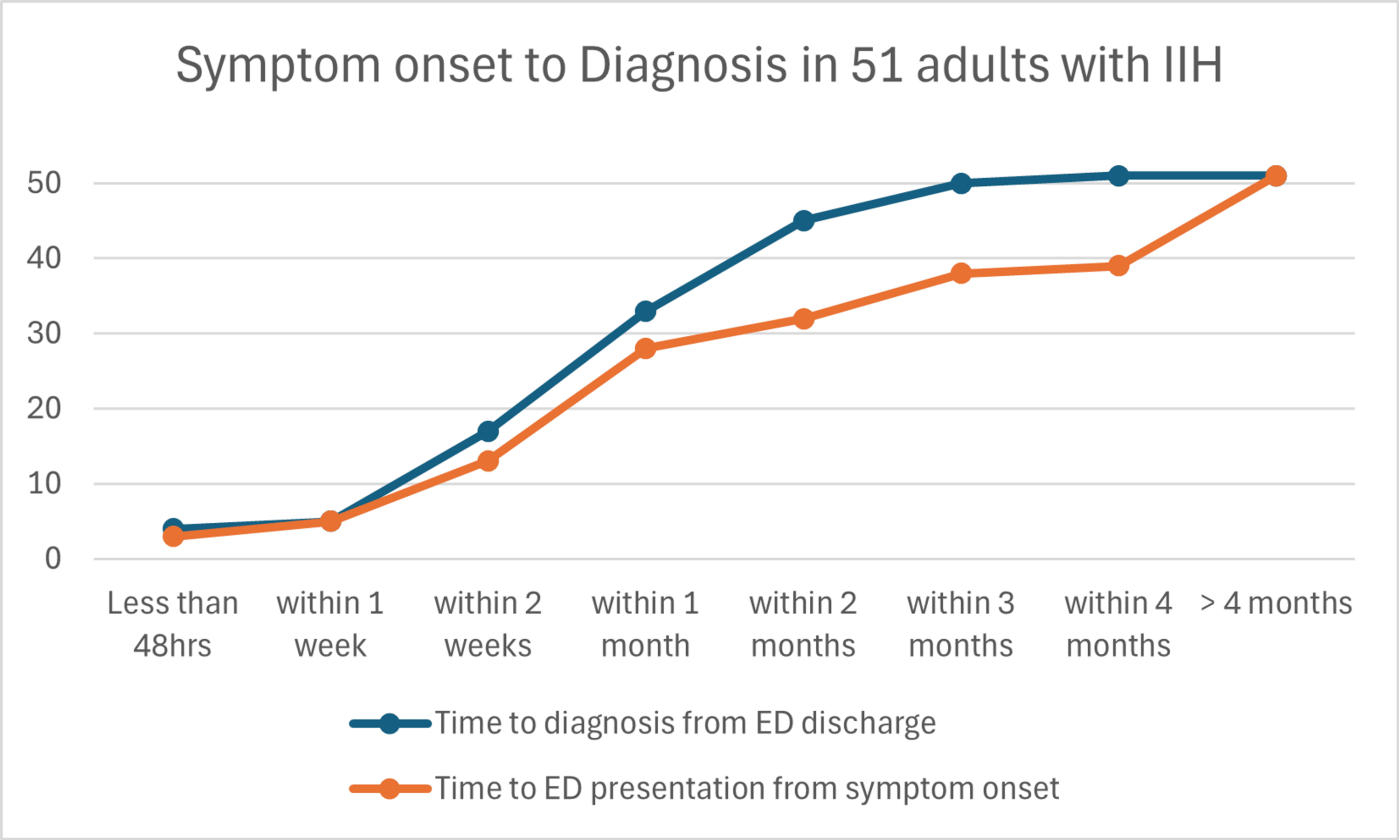
The cumulative number of patients presenting to the emergency department (ED) from initial symptom onset and the cumulative number of patients with a final diagnosis of idiopathic intracranial hypertension from ED discharge. IIH: idiopathic intracranial hypertension.

**Figure 3:**
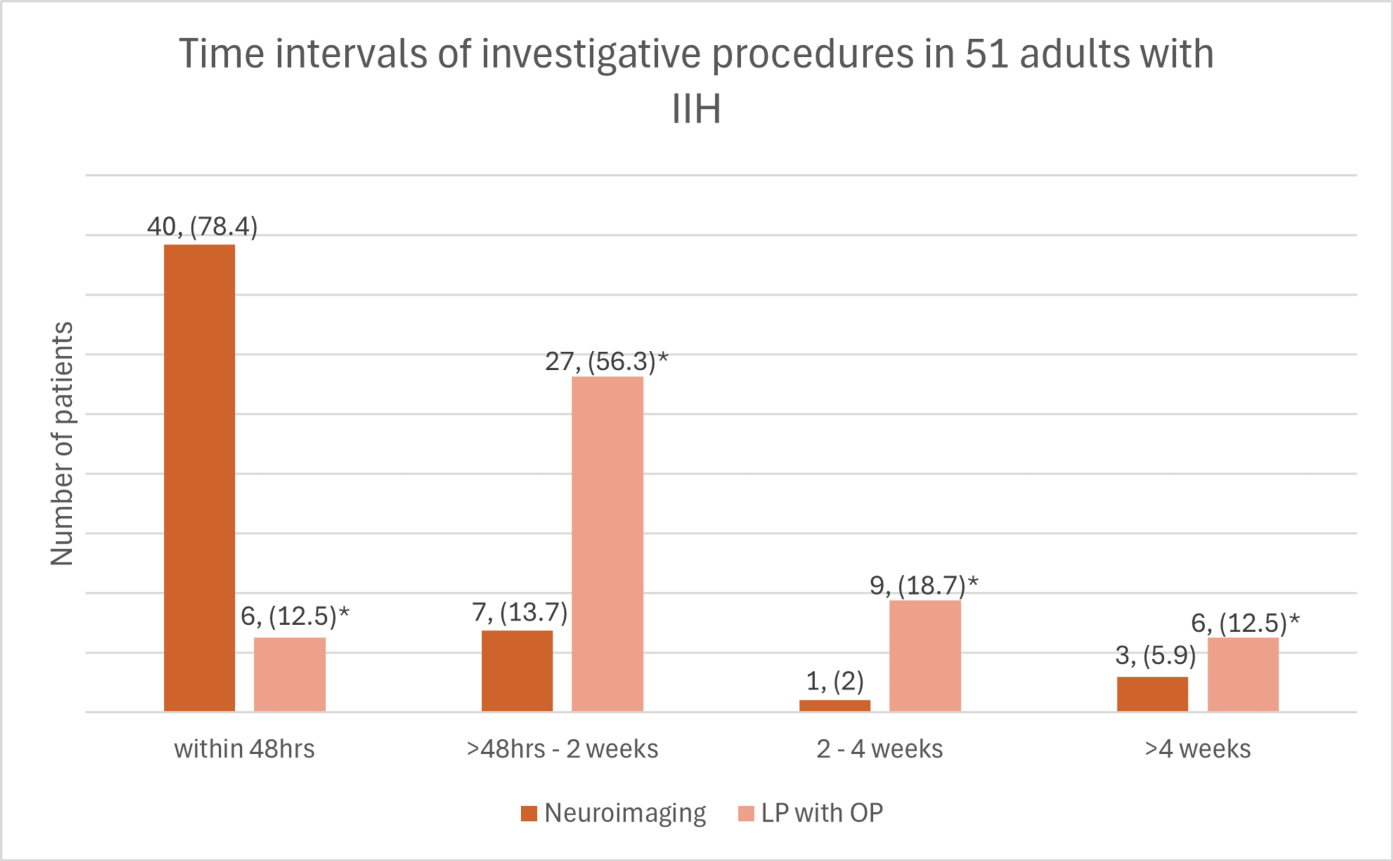
The rate of neuroimaging and lumbar puncture (LP) with opening pressure (OP). * The total number of adults who received a lumbar puncture with opening pressure was 48. IIH: idiopathic intracranial hypertension.

The time from initial symptoms to presentation to the ED for patients varied (Figure 2); 54.9% (28/51) presented within one month of symptoms commencing, 7.8% (4/51) presented within the second month, 11.8% (6/51) presented within the third month, and 25.5% (13/51) presented after three months. The average time from symptom onset to ED presentation was 132.2 days (range = 1-1767, SD = 283.8). Neuroimaging was performed during the ED stay (within 48 hours, including patients who had neuroimaging prior to ED presentation) in 78.4% (40/51) of patients, within two to 14 days since ED discharge in 13.7% (7/51), between two to four weeks in 2% (1/51), and after one month from ED discharge in 5.9% (3/51) of patients. The equivalent numbers for LP were 12.5% (6/48), 56.3% (27/48), 18.7% (9/48), and 12.5% (6/48), respectively. Three patients did not receive an LP; one was pregnant, in whom CT-guided LP was contraindicated, and two others were unable to tolerate the procedure. Tables 2, 3 and Figure 3 provide details regarding the timing and place of investigations (neuroimaging and LP) once seen in ED. A final diagnosis of IIH was achieved for 64.7% of patients within one month, and within four months for 100% of patients. The mean time to final IIH diagnosis from ED discharge was 31.1 days (range = 1-140, SD = 28.6). The rate of final diagnosis from ED discharge is presented in Figure 2. The mean time to final diagnosis from symptoms onset was 163.3 days (range = 11-1800, SD = 312.3). Timings for diagnosis are presented in Table 3.

**Table 2:** Geographic overview of investigations of idiopathic intracranial hypertension.

	Number	Percentage of patients, n = 51 (%)
CT brain with venogram		
In ED	28	55
Prior to ED	6	11
MRI		
In ED	5	10
Prior to ED	0	0
Outpatient	25	49
Had both an MRI and a CT	13	25
Lumbar puncture		
During ED stay	5	12
Prior to ED	1	0
Outpatient	38	88

**Table 3:** Temporal overview of investigations and diagnosis of idiopathic intracranial hypertension.

	Mean (SD, median)	Range
Time to CT in hours while in ED	5.9 (7.8, 5)	1-45
Time to MRI in days as an outpatient	19.28 (20.6, 8)	1-80
Time to lumbar puncture in days as an outpatient	16.6 (14.7, 9.4)	3-59
Time to diagnosis from symptom onset in days	163.3 (312.3, 67.0)	11-1800
Time to diagnosis from ED discharge in days	31.1 (28.6, 26.0)	1-140
Time to ED presentation from symptom onset in days	132.2 (283.8, 29.7)	1-1767

## Discussion

IIH is a neurological condition that has the potential to incur a high degree of morbidity as well as irreversible visual loss [1]. In this study, we investigated the demographics, investigations, and timeframe to diagnosis in 51 adult patients presenting with IIH to the RVEEH over a 12-month period. Due to the unique environment of a specialised ophthalmological ED, we anticipated a high number of patient presentations with new/undiagnosed IIH, which was consistent with our results. This is further highlighted when compared to a previous retrospective analysis from a general ED where a comparable number of patients with IIH were seen over a nine-year period [10]. Regarding patient demographics and clinical findings, our study is consistent with prior research. We observed a young female predominance, with many of our patients being identified as overweight [7,11,12]. Additionally, headache and affected vision were the most common presenting symptom for this patient group [13]. Interestingly, we did not observe any strabismus, vertigo, photophobia, or phonophobia. This may be attributed to gaps in clinical documentation or may highlight the rarity of these associated symptoms [14].

Diagnostic delay

A final diagnosis of IIH was achieved for 64% of patients within one month of ED presentation. The mean times to diagnosis from symptom onset and from ED discharge were 163.3 days and 31.1 days, respectively. The mean time to ED presentation from symptom onset was 132.2 days. These results show that the period prior to presenting to ED was on average more likely to cause a delay in diagnosis than the period post ED presentation. However, due to the retrospective nature of this study, the specific reasons for this delay were unable to be ascertained from the medical records. Causes for this delay may be attributed to the subtle and insidious onset of symptoms [8], challenging referral pathways to our institution as seen in other research [15], and limited awareness of this condition along with less experience in non-eye practitioners accurately identifying papilloedema among primary healthcare providers [16,17], resulting in a delay in referring to an eye specialist. Regardless, the overall delayed time to diagnosis from symptom onset emphasizes the need for high suspicion and appropriate referral for workup of this condition to enable an expedient diagnosis, especially in overweight young females with headache and visual symptoms. This is particularly prudent considering the importance that early treatment has in achieving a favourable visual outcome [5,12] as well as limiting the impact of disabling headaches [6].

Regarding the post-ED review, once a patient is initially seen by an eye specialist, they will on average have a diagnosis within 30 days. This time to diagnosis post initial specialist review is longer when compared to previous research. A similar retrospective analysis that studied the time to diagnosis in children and adolescents with IIH showed the majority of their patients (60%) had a diagnosis within 48 hours after their initial assessment from a specialist [7]. The relative lag to the final diagnosis in our cohort may be attributed to a few factors. Firstly, as many of our patients were given a diagnosis at outpatient follow-up (which is usually booked for two to four weeks from ED discharge), the time to final diagnosis is likely to be longer when compared to a system where patients can be seen earlier. Secondly, as our cohort was mainly adults, adherence to outpatient/diagnostic investigation appointments may be inferior (resulting in rebooking and delaying time to final diagnosis, which is observed in our patient group that received their outpatient investigations and clinic appointments later than the standard timeframe) than those of children/adolescents where their parents would likely ensure they would attend. Finally, our cohort waited longer on average to receive an LP with OP. LP with OP should be performed as soon as neuroimaging has excluded space-occupying lesions, Chiari malformation, and other contraindications [2]. However, due to the RVEEH not having interventional radiology/clinicians to perform LPs onsite, rapid LPs were not possible for most patients. This prevents patients from receiving a final diagnosis of IIH until this is performed, which is usually within two weeks of ED discharge. When comparing our rate of neuroimaging, it is equivalent to the paediatric study (74% performed in the first 48 hours vs. 73% performed in the first 48 hours, respectively), as such it is unlikely that our rate of neuroimaging is a factor in delaying diagnosis. Overall, it is likely that system factors were the main cause of delayed diagnosis in the post-ED review period.

Mitigating overdiagnosis

Overdiagnosis of IIH is common and can lead to individuals receiving unnecessary investigations and treatment [18]. This overdiagnosis has been attributed to inaccurate ophthalmological examination and reinforces the need for rapid access to specialists with experience in the diagnosis of optic nerve disorders [3]. Upon analysing and contrasting the rates of confirmed IIH diagnoses following specialist reviews, the accuracy in working diagnoses from our cohort (51/71, 72%) differs from previously published reports describing high rates of incorrect referrals (40% misdiagnosis rate) to other neuro-ophthalmology services [3]. Additionally, when reviewing our accuracy of identifying papilloedema on fundoscopy, only four patients (three diagnosed with migraine, one with functional vision loss) on review from a neuro-ophthalmologist were described as not having papilloedema/pseudopapilloedema (14 were observed to have pseudopapilloedema, which is a known mimic of papilloedema and challenging to differentiate, especially on fundoscopy [19]). Our accuracy in identifying papilloedema and improved diagnostic accuracy of IIH may be attributed to the unique positioning of the RVEEH, where the ED is equipped with ophthalmology consultants, on-call neuro-ophthalmologists, and registrars who have more clinical experience in ophthalmological examination skills compared to other referring practitioners. This is supported by the literature, which asserts an accurate ophthalmological examination by experienced providers, especially the recognition of papilloedema as an important factor in determining an accurate diagnosis of IIH [3,20].

Future considerations

Ideally, a structured rapid referral pathway would allow individuals with suspected IIH to be referred from a primary care physician or neurologist/headache specialist (as many of these patients present with headaches) to an optometrist/ophthalmologist where an accurate fundus examination can be performed. They would then be able to refer to a specialised neuro-ophthalmology centre (such as the RVEEH) if still concerned about IIH. This would ensure the patient has had an accurate diagnostic workup and limit the potential overdiagnosis of IIH. Encouraging collaboration between the specialities would likely improve the time delay to diagnosis. Such rapid referral pathways have been suggested before with an emphasis on an educational campaign around IIH in the primary and secondary healthcare system followed by a clear referral pathway to a specialised centre [15].

Limitations

There are several limitations to this study. First, there were many potential patients (n = 68) who were unable to be followed up (as they were seen at another healthcare facility with inaccessible health records). As such, this overall decreased the power of our study.

Secondly, our censoring regarding symptom onset relied on patient histories taken during their emergency department review, which often occurred weeks to months after symptom onset. Using this encounter to ascertain when symptom onset occurred rather than using medical records from their general practitioner or optometrist likely contributed to a less reliable date of symptom onset.

## Conclusions

Diagnosis of IIH can be difficult and is often delayed, usually due to a lag in being reviewed by an appropriate eye specialist. IIH should be considered in the differential diagnosis of young, overweight females with headaches, especially when visual symptoms are described. Furthermore, these patients should receive an accurate optic nerve examination by an experienced clinician to assist with diagnostic accuracy. While there were limited delays from initial contact with an eye specialist, our study highlights that a referral pathway to a specialist neuro-ophthalmology centre can result in a timely and accurate diagnosis for individuals suffering from IIH.
